# Exercise Improves Endothelial Function via the lncRNA MALAT1/miR-320a Axis in Obese Children and Adolescents

**DOI:** 10.1155/2021/8840698

**Published:** 2021-06-02

**Authors:** Wei Zhao, Yane Yin, Huiling Cao, Yandong Wang

**Affiliations:** ^1^Department of Neonatology, Weifang People's Hospital, Weifang 261041, Shandong, China; ^2^Department of Pediatrics, Weifang People's Hospital, Weifang 261041, Shandong, China

## Abstract

**Background:**

Endothelial dysfunction commonly occurs in obese children and adolescents, leading to an increased risk of cardiovascular diseases. Exercise has significant protective effects against endothelial dysfunction through regulating some noncoding RNAs. This study aimed to investigate the relationship of long noncoding RNA MALAT1 and microRNA-320a (miR-320a) with the exercise-induced improvement of endothelial dysfunction in obese children and adolescents.

**Methods:**

Sixty obese children and adolescents were included in this study, including 40 cases that received 12-week exercise training and 20 cases that received only diet control. The anthropometric and blood indices before and after exercise were recorded and compared, and the endothelial dysfunction was evaluated by examining the levels of markers, including VCAM-1, ICAM-1, and E-selectin, using an ELISA assay. The expression levels of noncoding RNAs were assessed using real-time quantitative PCR, and their correlation with patients' recorded indices and endothelial dysfunction markers was analyzed.

**Results:**

The 12-week exercise training significantly decreased the levels of VCAM-1, ICAM-1, and E-selectin and could inhibit MALAT1 but promote miR-320a expression in obese children and adolescents. The expression of MALAT1 and miR-320a was correlated with the changes in the anthropometric and blood indices of obese children and adolescents, and their correlations with endothelial dysfunction markers were obtained.

**Conclusion:**

All the data revealed that exercise has significantly protective effects against endothelial dysfunction and can regulate the expression of the MALAT1/miR-320a axis. MALAT1 and miR-320a were correlated with endothelial dysfunction markers, indicating that the MALAT1/miR-320a axis may be related with the alleviating effects of exercise on endothelial function in obese children and adolescents.

## 1. Introduction

Obesity is a disease in which long-term energy intake exceeds consumption, resulting in excessive energy stored in the form of fat and increased risk of diseases in various systems of the body [1]. Thus, obesity becomes one of the global public health burdens in both developed and developing countries, affecting the health and life quality of both adults and children [2]. Studies have shown that obesity in children and adolescents is an important risk factor for the onset of high blood pressure, diabetes, and cardiovascular diseases [3]. It is well known that normal endothelial function is one of the most critical mechanisms to maintain cardiovascular health [4]. Obese children and adolescents with endothelial dysfunction are at high risk with atherosclerosis and cardiovascular diseases [5]. Therefore, the effective evaluation and prevention of endothelial dysfunction are important approaches to prevent the development of cardiovascular diseases in obese children and adolescents.

Previous studies have reported that the levels of reactive oxygen species (ROS), C-reactive protein (CRP), and proinflammatory cytokines were significantly increased when endothelial dysfunction occurs, which led to the enhanced expression and secretion of some adhesion molecules, such as intercellular adhesion molecule 1 (ICAM-1) and vascular cell adhesion molecule 1 (VCAM-1), and E-, L-, and P-selections, and consequently promoting the concentration of blood and the formation of small patches [6, 7]. Therefore, VCAM-1, ICAM-1, and E-selectin are considered as important markers of endothelial dysfunction [8, 9]. Some studies have found that exercise significantly improves endothelial dysfunction caused by obesity, and preliminary possible mechanisms have been discussed [9, 10]. However, the current understanding of exercise-related mechanisms in the alleviation of obesity-associated endothelial dysfunction remains limited. Thus, there is an urgent need to further investigate the underlying mechanisms.

Metastasis-associated lung adenocarcinoma transcript 1 (MALAT1) is a widely investigated long noncoding RNA (lncRNA) and has been reported to be related with endothelial function [11–13]. MALAT1 expression has been found to be downregulated after exercise [14], and an important research has documented that MALAT1 participated in the protective effects of exercise against insulin resistance in type 2 diabetes mellitus [15]. In addition, a study by Sun et al. found that MALAT1 regulated endothelial biological function by sponging miR-320a [16]. Considering the dysregulation of MALAT1 after exercise and the role of the MALAT1/miR-320a axis in endothelial function, we deduced that the MALAT1/miR-320a axis might also play a potential role in the improving effects of exercise on endothelial function in obese children and adolescents.

In this study, the expression levels of MALTA1 and miR-320a in obese children and adolescents before and after exercise were examined, and their correlation with endothelial dysfunction was investigated by evaluating the levels of VCAM-1, ICAM-1, and E-selectin. The findings of this study may uncover the relationship between the MALAT1/miR-320a axis and the improved endothelial function induced by exercise.

## 2. Materials and Methods

### 2.1. Study Population

This study retrospectively analyzed the data from 60 obese children and adolescents (age range of 8–16 years) who were admitted to Weifang People's Hospital between May 2019 and August 2019. The following were inclusion criteria: (i) the body mass indexes (BMI) of the study objects were ≥23.9 kg/m^2^; (ii) none of the cases had cardiovascular diseases, kidney diseases, or chronic inflammation diseases; (iii) had no history of smoking and drinking; (iv) had no hormone medicine during the recent 6 months; and (v) had no physical activity restrictions or regular exercise habits. In addition, the cases with chronic diseases, endocrine diseases, dyskinesia, taking medications, smoking, allergies, thyroid diseases, cardiovascular diseases, and consumption of any supplement or oral antibiotics in previous 3 months were excluded. Only the data from children and adolescents who were in accord with the above criteria were collected and analyzed in this study. All the analyzed children and adolescents had BMI values more than or equal to the 95^th^ percentile. Informed consent was obtained from the parents of the participants, and the study procedures were approved by the Ethics Committee of Weifang People's Hospital.

### 2.2. Grouping and Exercise Training

Among the 60 obese children and adolescents, 40 cases (exercise group) were subjected to 12 weeks of diet control and exercise training and 20 cases (matched group) received diet control for 12 weeks, but still remained their previous life habits. For the diet control, all obese children and adolescents were required to appropriately reduce the intake of high-calorie and high-fat foods and increase the intake of vegetables and fruits. The carbohydrate ratio accounted for 40–50%, protein accounted for 20–30%, and fat accounted for 20–25%, and the intake of vitamins, trace elements, inorganic salts, and water was ensured. The energy intake was calculated based on the previously reported criteria [17, 18]. In addition, health education on nutrition knowledge was conducted weekly for obese children and adolescents. For exercise training, the aerobic exercise training included single jump rope, double jump rope, round-trip running, and aerobic gymnastics. The training was conducted 5 times per week, and each exercise lasted 50 min (10 min in preparation, 30 min in formal training, and 10 min in relaxation training). The exercise training was organized and managed by professionals, and the heart rate changes were detected by a heart rate monitor. The maximum heart rate was controlled at 60–70% during exercise.

### 2.3. Anthropometric Measurements

The value and BMI and circumferences of the neck, waist, and hip were measured and recorded before and after the 8-week of exercise training by the same individuals [19].

### 2.4. Blood Indices

After a 12-hour overnight fast, blood samples were collected from the participants before and after the intervention. Serum was isolated from the blood samples by centrifugation and stored at −20°C for further analyses. Total cholesterol (TC), triglycerides (TG), low-density lipoprotein cholesterol (LDL-C), high-density lipoprotein cholesterol (HDL-C), fasting blood glucose (FBG), and fasting insulin (FIN) were measured using an automatic biochemical analyzer (HITACHI 7600, Japan). The homeostasis model of assessment of insulin resistance (HOMA-IR) was calculated.

### 2.5. Endothelial Dysfunction Marker Detection

The levels of endothelial dysfunction markers in serum samples, including VCAM-1 (Cat. # DVC00), ICAM-1 (Cat. # DCD540), and E-selectin (Cat. # DSLE00), were measured using enzyme-linked immunosorbent assay (ELISA) kits (R&D System, MN, USA) following the manufacture's protocols.

### 2.6. RNA Extraction and Real-Time Quantitative PCR (RT-qPCR)

Total RNA was extracted from serum samples using TRIzol reagent (Invitrogen, Carlsbad, CA, USA). cDNA was synthesized from RNA using a PrimeScript RT reagent kit (TaKaRa, Shiga, Japan) following the manufacturer's instruction. The relative expression of MALAT1 and miR-320a was measured using qPCR with SYBR green I Master Mix kit (Invitrogen, Carlsbad, CA, USA) on a 7500 Real-Time PCR System (Applied Biosystems, USA). GAPDH and U6 were used as internal controls for MALAT1 and miR-320a, respectively. The method of 2^−ΔΔCt^ was used to calculate the final expression values.

### 2.7. Statistical Analysis

SPSS 26.0 (SPSS Inc., Chicago, IL) and GraphPad 7.0 were (GraphPad Software, Inc., USA) were used to analyze the data of this study. The data were expressed as mean ± SD, and differences were compared using Student's *t*-test and one-way ANOVA. The Pearson correlation method was used for correlation analysis. A *P* < 0.05 was considered statistically significant.

## 3. Results

### 3.1. Baseline Characteristics of the Participates before and after Intervention

The anthropometric data and blood data of the obese children and adolescents before and after the dietary and/or exercise intervention are given in Table 1. Compared to the matched group, in which the obese children and adolescents received only dietary intervention, the exercise group had no significant differences in age, gender, BMI, circumferences of neck, waist and hip, TC, TG, LDL-C, HDL-C, or HOMA-IR before intervention (all *P* > 0.05). After exercise training, BMI, circumferences of neck, waist and hip, TC, TG, LDL-C, and HOMA-IR were significantly decreased in obese children and adolescents in the exercise group (all *P* < 0.05). In addition, regarding the data obtained after the intervention, the obese children and adolescents in the exercise group had lower BMI, circumferences of the neck, waist, and hip, TC, TG, LDL-C, and HOMA-IR and higher HDL-C compared to the cases in the matched group, and the differences in BMI, circumferences of waist and hip, TC, LDL-C, and HOMA-IR are statistically significant (all *P* < 0.05).

### 3.2. Changes of Endothelial Dysfunction Markers in Obese Children and Adolescents

The levels of endothelial dysfunction markers, including VCAM-1, ICAM-1, and E-selectin, were measured using ELISA assay, and the results shown in Table 2 revealed that there were no differences in the markers before the intervention of this study between the matched and exercise groups (all *P* > 0.05). The levels of endothelial dysfunction markers after the intervention showed a slight decrease in obese children and adolescents of the matched group, but the differences did not reach statistical levels (all *P* > 0.05). Of note, the levels of VCAM-1, ICAM-1 and E-selectin were significantly decreased in obese children and adolescents after 12-week exercise training (all *P* < 0.05). In comparison with the detection results in the matched group after intervention, the obese children and adolescents in the exercise group had significantly decreased levels of VCAM-1, ICAM-1, and E-selectin (all *P* < 0.05). All these data indicated that exercise could significantly improve endothelial function.

### 3.3. Expression of the MALAT1/miR-320a Axis in Obese Children and Adolescents

**Figure 1:**
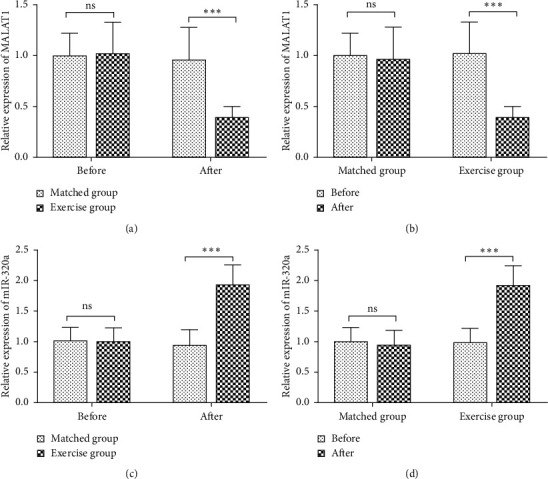
Relative expression of MALAT1 and miR-320a in obese children and adolescents before and after intervention. (a) There was no difference in MALAT1 expression between the matched group and the exercise group, but 12-week exercise training in the exercise group significantly reduced the expression of MALAT1 compared to the matched group receiving only dietary control. (b) After exercise training, the expression of MALAT1 was decreased in obese children and adolescents. (c) There was no difference in miR-320a expression between the matched group and the exercise group, but 12-week exercise training in the exercise group significantly increased the expression of miR-320a compared to the matched group receiving only dietary control. (d) After the exercise training, the expression of miR-320a was elevated in obese children and adolescents. ns, no significance;  ^*∗∗∗*^*P* < 0.001.

The expression of MALAT1 and miR-320a measured by RT-qPCR is shown in Figure 1. The results in Figures 1(a) and 1(b) indicated that there was no difference in the expression of MALAT1 and miR-320a in obese children and adolescents between the matched group and exercise group before intervention (both *P* > 0.05). After the diet and/or exercise training, MALAT1 expression was significantly decreased, and miR-320a expression was markedly increased in the exercise group compared with that in the matched group (both *P* < 0.05). Furthermore, by analyzing the differences before and after the intervention, obese children and adolescents had significantly downregulated MALAT1 expression and upregulated miR-320a expression after 12-week exercise, while the changes in MALAT1 and miR-320a were not significant in the obese children and adolescents receiving only dietary intervention (both *P* > 0.05) (Figures 1(c) and 1(d)).

### 3.4. Relationship between the MALAT1/miR-320a Axis and the Baseline Characteristics of Obese Children and Adolescents after Exercise

Correlations of MALAT1/miR-320a axis with the anthropometric features and blood indices after exercise were evaluated by the Pearson correlation method. The data listed in Table 3 show that serum MALAT1 levels were positively correlated with BMI, circumferences of waist and hip, TC, LDL-C, and HOMA-IR (all *P* < 0.05), while serum miR-320a expression was negatively correlated with BMI, waist circumference, TC, FBG, LDL-C, and HOMA-IR (all *P* < 0.05).

### 3.5. Correlation of the MALAT1/miR-320a Axis with Endothelial Dysfunction after Exercise

**Figure 2:**
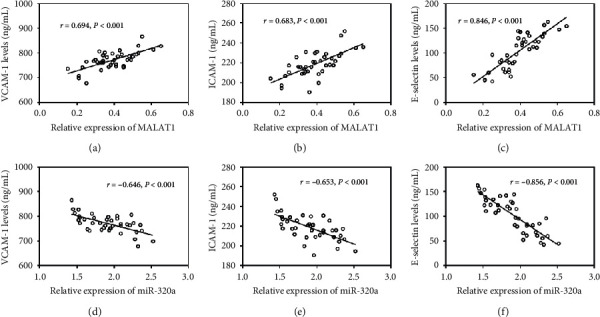
Correlation of MALAT1 and miR-320a levels with the levels of endothelial dysfunction markers. (a)–(c). The expression of MALAT1 was positively correlated with the levels of VCAM-1, ICAM-1, and E-selectin (all *P* < 0.001). (d)–(f). The expression of miR-320a was negatively correlated with the levels of VCAM-1, ICAM-1, and E-selectin (all *P* < 0.001).

To evaluate the correlation between changes in MALAT1/miR-320a axis and endothelial dysfunction after exercise in obese children and adolescents, the correlations of MALAT1 and miR-320a with VCAM-1, ICAM-1, and E-selectin levels were assessed. As shown in Figure 2, MALAT1 was positively correlated with the levels of VCAM-1, ICAM-1, and E-selectin (all *P* < 0.001), whereas miR-320a expression was negatively correlated with VCAM-1, ICAM-1, and E-selectin levels (all *P* < 0.001).

## 4. Discussion

The beneficial effects of exercise have been reported in various human diseases, especially in cardiovascular disorders [20, 21]. Exercise can significantly reduce the risk of cardiovascular diseases [22]. A study by Yang et al. showed that physical exercise acts as a potential medicine for atherosclerosis, and this study summarizes the mechanisms and clinical applications of exercise [23]. The quality of life and prognosis of patients with heart failure can be improved by exercise training, and the American College of Cardiology/American Heart Association, European Society of Cardiology, and National Institute for Health and Care Excellence (NICE) have recommended exercise-based cardiac rehabilitation as an adjunct for heart failure patients with unlimited exercise [24]. High-intensity interval exercise training (HIIT) and continuous exercise training could improve postexercise heart rate recovery in patients with chronic ischemic heart disease, and the beneficial effects of HIIT were more obvious [25]. The regulation of endothelial function by exercise plays an important role in the mechanisms of exercise improving cardiovascular diseases [26]. Endothelial dysfunction commonly occurs in obese children and adolescents, leading to an increased risk of cardiovascular diseases [5]. In obese adolescents, exercise has been documented to alleviate cardiovascular disorders by regulating miR-126 [27]. In this study, we also observed the beneficial effects of exercise in obese children and adolescents, which manifested by significantly reduced BMI, circumferences of the neck, waist, and hip, and low levels of TC, TG, LDL-C, and HOMA-IR. More importantly, the levels of VCAM-1, ICAM-1, and E-selectin, which are three major markers of endothelial dysfunction [9], were remarkably decreased in obese children and adolescents after a 12-week exercise training, which indicated the protective effects of exercise on endothelial dysfunction in obese children and adolescents.

Despite, it is well known that exercise can improve physical health, and the underlying mechanisms of exercise remain unclear. Emerging studies have emphasized the association of noncoding RNAs with exercise. For example, Liu et al. reported some lncRNAs that could be regulated by exercise training in insulin resistance-related vascular injury and proposed that the deregulated lncRNAs might be biomarkers to reflect vascular injury in high-fat diet-induced insulin resistance [28]. The antidiabetic and antidepression effects of Baduanjin exercise have been documented to be associated with some aberrantly expressed lncRNAs and circRNAs [29]. Increased expression of miR-17-3p has been found to enhance the protective effects of exercise on myocardial ischemia-reperfusion injury [30]. miR-222 can be upregulated by exercise and mediates the beneficial effects of exercise on pathological cardiac remodeling [31]. These previous studies suggested that exercise may exert protective effects through regulating noncoding RNAs.

MALAT1 is a widely studied lncRNA, and its regulatory effects on cellular functions have been reported in different cell types [32, 33]. MALAT1 has been reported to regulate endothelial cell function and vessel growth and to be involved in the endothelial dysfunction in diabetes mellitus [11, 12]. More importantly, it is an lncRNA that can be regulated by exercise. Paronetto et al. documented the relationship between MALAT1 and the prevention of cancer by exercise [14]. Liu et al. found the mediating role of MALAT1 in the alleviating effect of exercise on insulin resistance in type 2 diabetes mellitus [15]. MALAT1-mediated inhibition of apoptosis plays an important role in the cognitive improvement by exercise in ischemia/reperfusion injury [34]. In the present study, we found the decreased levels of MALAT1 in obese children and adolescents after exercise, and exercise-induced changes in MALAT1 expression were positively correlated with the levels of endothelial dysfunction markers. Thus, we considered that the downregulated MALAT1 might be related with the improved endothelial function induced by exercise.

miR-320a has been identified as a target of MALAT1 and participates in the regulation of endothelial function by MALAT1 [16, 35]. In this study, the expression patterns of miR-320a were significantly upregulated by exercise training in obese children and adolescents, which was opposite to that of MALAT1 after exercise. Similar to the correlation results of MALAT1 with markers of endothelial dysfunction, we also found the correlation of miR-320a with endothelial dysfunction markers. Considering the reported directly regulatory relationship between MALAT1 and miR-320a, we considered that the MALAT1/miR-320a axis might be associated with the improved endothelial function induced by exercise in obese children and adolescents.

Taken together, the data of this study confirmed the protective effects of exercise on endothelial dysfunction in obese children and adolescents and demonstrated that exercise training significantly reduces MALAT1 expression and increases miR-320a expression and that the MALAT1/miR-320a axis may be related with the alleviating effects of exercise on endothelial function in obese children and adolescents with obesity. The findings of this study further uncover the mechanisms underlying the beneficial effects of exercise on endothelial dysfunction, and the MALAT1/miR-320a axis may provide novel targets for the improvement of endothelial function in obese children and adolescents. This study have some limitations, which mainly include the limited sample size and the lack of verification using animal models. Further investigations are necessary to confirm the conclusion of this study using a larger study population. In addition, in vitro experiments using endothelial cells and obese animal models should be carried out to study the role of the MALAT1/miR-320a axis in the mechanisms of exercise.

## Figures and Tables

**Table 1:** Comparisons of the baseline characteristics of obese children and adolescents before and after intervention.

Characteristics	Matched group (*n* = 20)		Exercise group (*n* = 40)	
	Before	After	Before	After
Age (years)	12.05 ± 2.28	12.05 ± 2.28	12.30 ± 2.61^ns^	12.30 ± 2.61
Gender (males/females)	13/7	13/7	28/12^ns^	28/12
BMI (kg/m^2^)	29.33 ± 1.11	28.63 ± 0.94	29.33 ± 1.29^ns^	26.09 ± 1.05 ^*∗*^ ^#^
Neck circumference (cm)	39.30 ± 3.99	39.10 ± 2.49	40.50 ± 4.16^ns^	36.80 ± 2.71 ^*∗*^
Waist circumference (cm)	93.00 ± 2.24	87.50 ± 1.90	92.20 ± 2.65^ns^	78.10 ± 2.85 ^*∗*^ ^#^
Hip circumference (cm)	99.40 ± 2.67	96.20 ± 2.86	99.60 ± 2.55^ns^	90.80 ± 2.88 ^*∗*^ ^#^
TC (mM)	4.86 ± 0.26	4.06 ± 0.17	4.82 ± 0.19^ns^	3.44 ± 0.16 ^*∗*^ ^#^
TG (mM)	1.73 ± 0.27	0.90 ± 0.08	1.79 ± 0.22^ns^	0.77 ± 0.06 ^*∗*^
LDL-C (mg/dL)	91.77 ± 13.90	89.85 ± 13.76	92.64 ± 14.30^ns^	85.08 ± 13.60 ^*∗*^ ^#^
HDL-C (mg/dL)	46.44 ± 9.04	49.72 ± 8.30	48.78 ± 9.58^ns^	51.12 ± 5.70
HOMA-IR	3.17 ± 1.55	3.01 ± 1.42	3.34 ± 1.63^ns^	2.51 ± 0.97 ^*∗*^ ^#^

BMI, body mass index; TC, total cholesterol; TG, triglyceride; LDL-C, low-density lipoprotein cholesterol; HDL-C, high-density lipoprotein cholesterol; HOMA-IR, homeostatic model assessment-insulin resistance; ns, no significance when compared to the data before intervention in the matched group.  ^*∗*^*P* < 0.05 when compared to the data before intervention in the exercise group. #*P* < 0.05 when compared to the data after intervention in the matched group.

**Table 2:** Endothelial dysfunction markers in obese children and adolescents before and after intervention.

Indicators	Matched group (*n* = 20)		Exercise group (*n* = 40)	
	Before	After	Before	After
VCAM-1 (ng/mL)	793.23 ± 19.16	782.21 ± 24.97	792.96 ± 36.00^ns^	770.88 ± 36.13 ^*∗*^
ICAM-1 (ng/mL)	245.91 ± 14.92	239.00 ± 15.15	242.30 ± 17.53^ns^	218.58 ± 12.92 ^*∗*^ ^*#*^
E-selectin (ng/mL)	169.90 ± 77.77	143.76 ± 54.20	171.05 ± 60.59^ns^	101.40 ± 37.54 ^*∗*^ ^*#*^

VCAM-1, vascular cell adhesion molecule 1; ICAM-1, intercellular adhesion molecule 1; ns, no significance when compared to the data before intervention in the matched group.  ^*∗*^*P* < 0.05 when compared to the data before intervention in the exercise group.  ^#^*P* < 0.05 when compared to the data after intervention in the matched group.

**Table 3:** Correlation of the MALAT1/miR-320a axis with the baseline features of obese children and adolescents after exercise.

Characteristics	MALAT1 expression		miR-320a expression	
	*R* value	*P* value	*R* value	*P* value
Age	0.001	0.982	−0.008	0.962
Gender	0.090	0.579	−0.027	0.867
BMI	0.325	0.031	−0.361	0.029
Neck circumference	0.092	0.171	−0.175	0.280
Waist circumference	0.213	0.043	−0.226	0.041
Hip circumference	0.289	0.038	−0.267	0.040
TC	0.452	0.012	−0.501	0.008
TG	0.121	0.246	−0.128	0.330
LDL-C	0.512	0.008	−0.499	0.010
HDL-C	−0.188	0.231	0.143	0.379
HOMA-IR	0.446	0.025	−0.431	0.028

BMI, body mass index; TC, total cholesterol; TG, triglyceride; LDL-C, low-density lipoprotein cholesterol; HDL-C, high-density lipoprotein cholesterol; HOMA-IR, homeostatic model assessment-insulin resistance.

## Data Availability

The data used to support the findings of this study are available from the corresponding author upon request.
